# Microstructure and Mechanical Properties of Laser Welded Al-Si Coated Hot-Press-Forming Steel Joints

**DOI:** 10.3390/ma12203294

**Published:** 2019-10-11

**Authors:** Xi Chen, Zhenglong Lei, Yanbin Chen, Meng Jiang, Ze Tian, Jiang Bi, Sanbao Lin

**Affiliations:** State Key Laboratory of Advanced Welding and Joining, Harbin Institute of Technology, Harbin 150001, China; chenxi_hit2016@163.com (X.C.);

**Keywords:** Al-Si coated HPF steel, laser welding, microstructure, mechanical properties

## Abstract

High strength steel has attracted a lot of attention due to its excellent advantage of weight reduction. A thin Al-Si coating covered on the surface of hot-press-forming (HPF) steel offers functions of antioxidation and decarburization under high temperature processing conditions. In this study, the microstructure characteristic, phase, microhardness, and tensile strength of laser welded Al-Si coated HPF steel joints were investigated at different laser powers. Experimental results show that the welding process becomes unstable because of metallic vapor generated by ablation of the coating. Some of the white bright rippled Fe-Al phase was observed to be distributed in the fusion zone randomly. It is found that microhardness, tensile strength, and cupping test qualification rate decreases with the increase of the laser power. For the 1.1 kW laser power, the sound weld performs the best mechanical properties: Microhardness of 466.53 HV and tensile strength of 1349.9 MPa.

## 1. Introduction 

Based on ensuring the strength and safety performance of vehicles, light weight of automobile can reduce the weight of overall vehicle as far as possible, so as to improve the power of the car and reduce fuel consumption and exhaust pollution. Advanced high strength steel (AHSS) and ultra-high strength steel (UHSS) are now being used for reinforcing car bodies increasingly in the automotive industry [1]. It is noted that due to the insufficient ductility, a hot press forming process is proposed and developed to avoid spring back behavior of the high strength steel and ensure the formability quality [2,3,4]. The interesting tensile strength of the processed component can reach as high as 1500 MPa approximately. However, the hot stamping process brought some new problems such as surface oxidation, scaling, corrosion, and wear [5,6]. The Al-Si coated high strength steel emerges at the moment to solve these problems [7]. 

Some previous researches have given the results that the Al-Si coating can be diluted into the weld zone during the welding process of the Al-Si coated steel. It results in the formation of the Fe-Al intermetallic phase and solid solutions. T.J. Park et al. investigated the effect of Al–8% Si coating layer on the hardness of the GTA welded ferritic stainless steels and found that the hardening of the fusion zone was attributed to the solid solution strengthening [8]. T.J. Yoon et al. analyzed the microstructure and phase transformation in the laser welded fusion zone before and after hot stamping heat treatment. Two types of Al segregation were observed to be distributed unevenly in the fusion zone with microstructures of martensite and bainite and formed along the fusion boundary with microstructure of Fe_3_(Al, Si) [9]. The Al content in the segregation zone affects the ferrite formation in the fusion boundary and zone.

Currently, laser welding as a joining technology has been applied more and more [10,11,12,13,14]. In the field of vehicle, laser welding, laser-arc hybrid welding, and laser welding-brazing of car parts have attracted a lot of attention [15,16,17,18]. In recent years, only a few relevant studies focus on laser welding of advanced and ultra-high strength steel for automotive application. J.K. Choi et al. has found that degradation of laser weldments has more significant influence on an overlap welded joint than a butt welded one [19]. C. Kim et al. has concluded that partially developed intermetallic phase precipitated along the fusion line weakened the strength of the joint. Aluminum and silicon were dissolved into the joint and formed the solid solution. The phase transformation analysis results are consistent with that of T.J. Yoon [20]. Besides, some other researchers ArcelorMittal Co. [21], J.H. Moon et al. [22], and F. Li et al. [23] reported that laser ablation technology could remove the Al-Si coating before welding. However, other researchers proposed that it might not be an ideal method due to the extra steps required for laser ablation. It is a somewhat controversial solution [24,25]. 

In general, previous researches mainly focused on the influence of with and without Al-Si coating as well as before and after hot stamping on laser welded joints. In this study, laser welding was conducted on Al-Si coated hot-press-forming (HPF) steels at different laser powers. The laser welding characteristics of the Al-Si coated HPF steels without heat treatment were investigated including the microstructure characteristics, phase, microhardness, and tensile strength of the welded joints. 

## 2. Experimental Procedures

### 2.1. Materials

**Figure 1:**
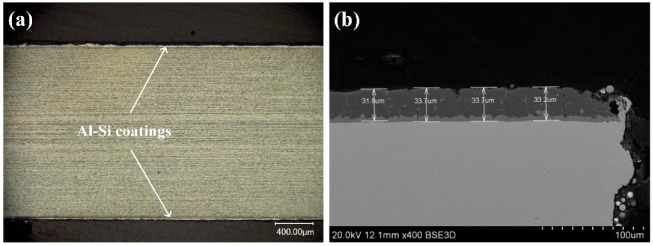
Cross-section morphologies of Al-Si coated hot-press-forming (HPF) steel: (**a**) Optical image and (**b**) SEM image.

The materials used in this study were HPF steel, and boron steel, with a total thickness of 2 mm. The base plates were coated with Al-Si coatings on both sides. Figure 1 shows the cross-sectional images of the base metal with two different contrast layers (Figure 1). The top layer was the Al-Si layer with a thickness between 30–40 μm. The intermetallic layer adjacent to the base metal was the Fe-Al intermetallic compound with a thickness of about 10 μm. The boron steel was composed of ferrite and pearlite. 

### 2.2. Welding Process

After installing the samples on the welding fixture, the indicator light was adjusted to the appropriate position for the next step. A continuous fiber laser (YLS-10000, IPG, Oxford, MA, USA) with a maximum output power of 10 kW was used to conduct the single-laser butt welding process. A six-axis KUKA robot was used to carry the laser head to implement the laser welding path. The diameter of the laser beam focused on the base materials was about 200 μm. The laser welding process parameters are listed in Table 1. A high speed camera (CamRecord 5000X2, Optronis, Kehl, Bade-Wurtemberg, Germany) with a maximum sampling frequency of 5000 f/s was used for capture laser welding behavior during the process. The auxiliary light source was generated by a semiconductor pulse laser (CAVILUX HF, CAVITAR, Tampere, Finland) with a wavelength of 808 nm. 

### 2.3. Microstructures and Chemical Compositions

Metallographic cross-sectional morphologies and weld size of the joints were observed and measured by optical microscopy (PMG3, Oympus, Tokyo, Japan). Mixed solution alcohol (98 vol.%) and nitric acid (2 vol.%) were used as etching reagents to reveal microstructures of the joints. Scanning electron microscopy (S-4700, Hitachi, Tokyo, Japan) was used to reveal microstructure characteristics. Energy dispersive spectrum fitted to the scanning electron microscopy was utilized to analyze the elemental compositions of different areas in the butt joint. Transmission electron microscopy (Tecnai G2 F30, FEI, Hillsboro, OR, USA) and focused ion beam (Helios NanoLa 600, FEI, Hillsboro, OR, USA) were applied for further microstructure investigation. 

### 2.4. Mechanical Measurements

Hardness profile can be obtained by using a Vickers micro-hardness tester (HVS-50, TBT, Nanjing, China) with a load of 100 g and a dwell time of 10 s. The micro-hardness of the fusion zone, HAZ, and featured zone were all tested. The indentation points were spaced at 0.15 mm and the profiles were obtained across the cross-sectional joint for all the samples. 

**Figure 2:**
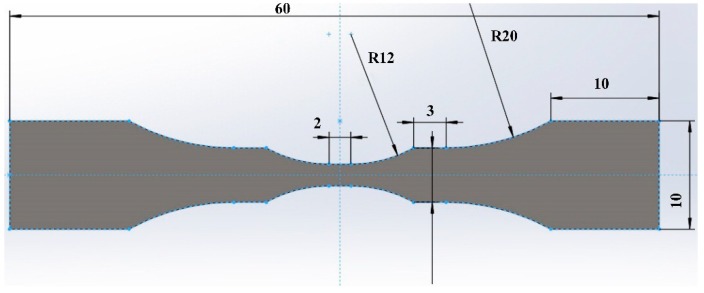
Schematic diagram of the non-standard tensile specimen.

Tensile strength tests were performed by universal material testing machine (5569, Instron, Norwood, MA, USA) at a cross-head speed of 1 mm/min at room temperature. Since the HPF steel used in this work had not been heat treated, thus the tensile strength of the base material was much lower than that of the joint. In order to obtain the tensile property of the joint, non-standard sized samples were designed to achieve the tensile of the joint in this study, as shown in Figure 2. 

## 3. Results and Discussion 

### 3.1. Weld Appearances

**Figure 3:**
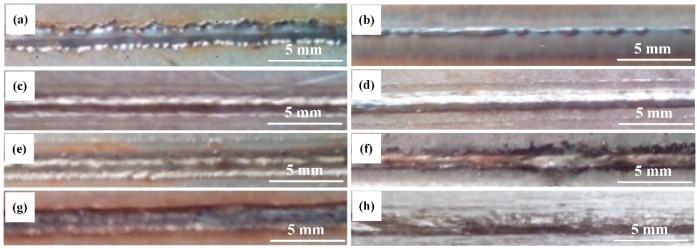
Weld appearance of HPF steels with Al-Si coatings: (**a**) Front at 0.9 kW laser power, (**b**) back at 0.9 kW laser power, (**c**) front at 1.1 kW laser power, (**d**) back at 1.1 kW power, (**e**) front at 1.8 kW laser power, (**f**) back at 1.800 W laser power, (**g**) front at 2.7 kW laser power, and (**h**) back at 2.7 kW.

To investigate the appropriate welding conditions initially, Figure 3 shows the front and back images of welding bead with different laser powers at a fixed welding speed of 1.5 m/min and a defocusing distance of −1 mm. It could be found that the back of weld was not continuous without full penetration for 0.9 kW laser power. With the increasing of the laser power, weld reinforcement at back of the weld bead increased overall. When the laser power was 1.1 kW, the weld realized fully penetration with a sound front and back appearance. 

**Figure 4:**
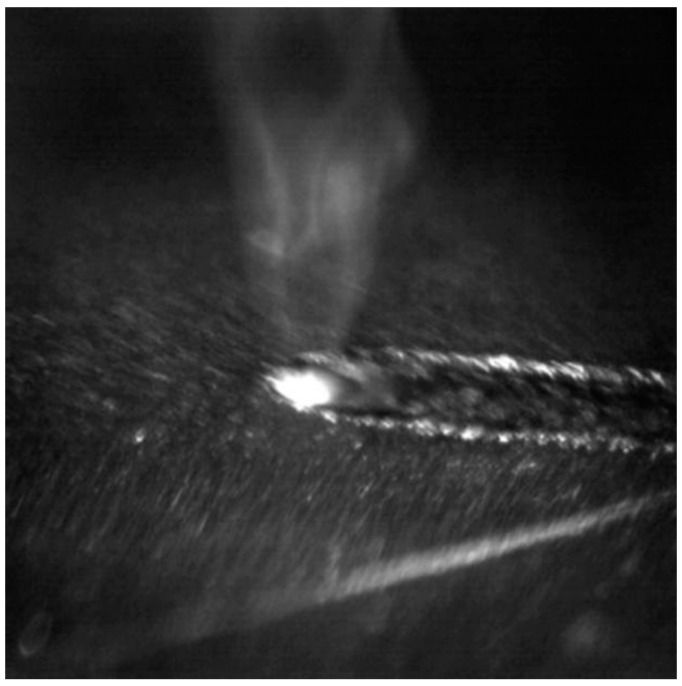
A representative high speed camera image of laser welding of Al-Si coated HPF steel at the laser power of 1.1 kW.

Figure 4 shows that there was apparent white metal vapor above the melting pool during laser welding process. The characteristics of the exhibited metal vapor effect were almost the same at all laser power. This is because the high energy density laser beam caused the Al-Si coating to melt and evaporate. Some metal vapors formed by vaporized Al and Si are shown in Figure 4. While the others were involved into the fusion zone with flow of welding pool and reacted with Fe. The generated intermetallic compounds or solid solutions would be given further discussion in the following analysis. 

**Figure 5:**
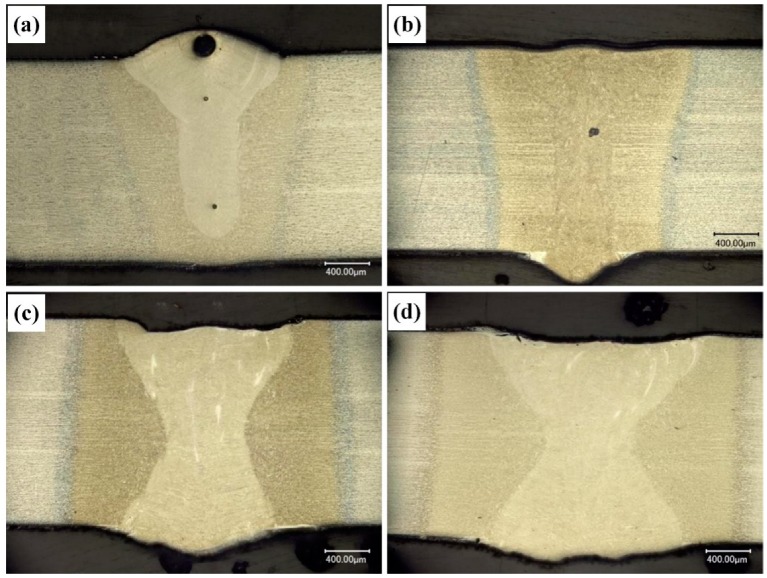
Cross-sectional shapes of welding joints at different laser powers: (**a**) 0.9 kW, (**b**) 1.1 kW, (**c**) 1.8 kW, and (**d**) 2.7 kW.

Laser welding can be divided into two types, namely laser deep penetration welding and laser conduction welding according to the existence of keyhole effect. Enough high energy density laser can support the formation of keyhole and maintain stability. In addition, different welding process parameters have considerable impacts on the shape of the keyhole. There were complicated interactions between the plasma and laser. The cross-sectional shapes of the joints at different laser powers are shown in Figure 5. It can be found that for 0.9 kW, the weld performed incomplete fusion penetration “I” type shape with 1.72 mm deep, while the weld shows a full penetration “I” type shape for 1.1 kW laser power. As for laser powers of 1.8 kW and 2.7 kW, the widths of both weld pool and HAZ increased with the increase of laser power. Table 2 displays the detailed size of the welds. 

### 3.2. Crystallization Behavior and Microstructure Characteristics of the Joints 

In general, crystallization starts at the boundary of weld pool in the process of welding. Non-spontaneous nucleation occurs on the surface of base metal grains of the specimens, which grows from the base metal grain to the middle of the weld pool in the form of columnar crystal, and then becomes epitaxial solidification. Since the grains of base metal grow into columnar crystals in the weld zone, the grain size of weld zone was similar to that of the base metal near the fusion line at the beginning of crystallization. After the thermal cycle, the base metal grains tended to be thick when heated, and the columnar crystals in the weld were also prone to becoming thick and large.

**Figure 6:**
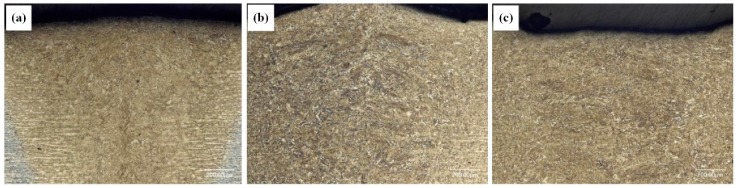
200× metallographic photos of welds obtained at different laser power: (**a**) 1.1 kW, (**b**) 1.8 kW, and (**c**) 2.7 kW.

Figure 6 shows the 200× metallographic photos of the welds obtained at the parameters listed in Table 1. Due to the different distances from the welds to the laser during welding, the thermal cycles the welds experienced were also different. Since the heating temperature in the welding process exceeded the austenite conversion temperature A_c3_, and the laser welding process was a rapid heating–cooling process, thus the obtained welds were dominated by martensite. As can be seen from in Figure 6, the microstructures of the three kinds of specimens were basically the same. All the microstructures were basically composed of columnar crystals with a small number of needle-like martensites distributed along the grain boundary of columnar crystals. The columnar crystals were mainly composed of massive martensites.

**Figure 7:**
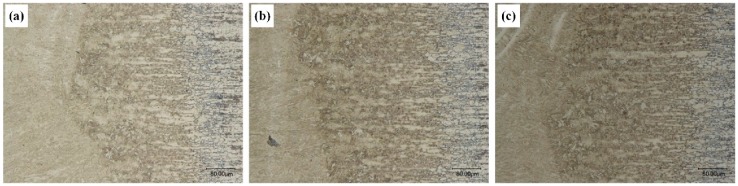
500× metallographic photos of heat-affected zones obtained at different laser power: (**a**) 1.1 kW, (**b**) 1.8 kW, and (**c**) 2.7 kW.

As can be seen from the HAZ metallographic microstructures of the three kinds of specimens in Figure 7, the HAZs of the three kinds of specimens were basically the same. From left to right, the HAZ was comprised of the quenching zone, incomplete quenching zone, and tempered zone. The coarse grain zone near the quenching zone close to the fusion line were basically rough needle-like and block-like martensites and granular bainites, as well as some pearlites. The quenching zone in the hot affected area was basically fine-grained martensites and a small number of fine lamellar pearlites. The incomplete quenching zone was martensites and perlites produced by phase transformation. In the tempered zone, the temperature was lower than A_c1_ during the entire welding process. The complete austenitizing temperature was not reached, and the microstructure was mainly ferrites and pearlites. Meanwhile, ferrites still remained the state of base metal basically, and showed zonal distribution. Martensite was the supersaturated solid solution of C element in α-Fe, and a large number of defects, such as dislocation, twin crystals, etc. exist in martensite. Therefore, the existence of martensite was unstable and easy to be decomposed when heated. During the welding process, the martensites in the incomplete quenching zone were prone to undergo tempering transformation and produced some carbide.

### 3.3. Chemical Compositions and Phase Analysis of the Joints 

**Figure 8:**
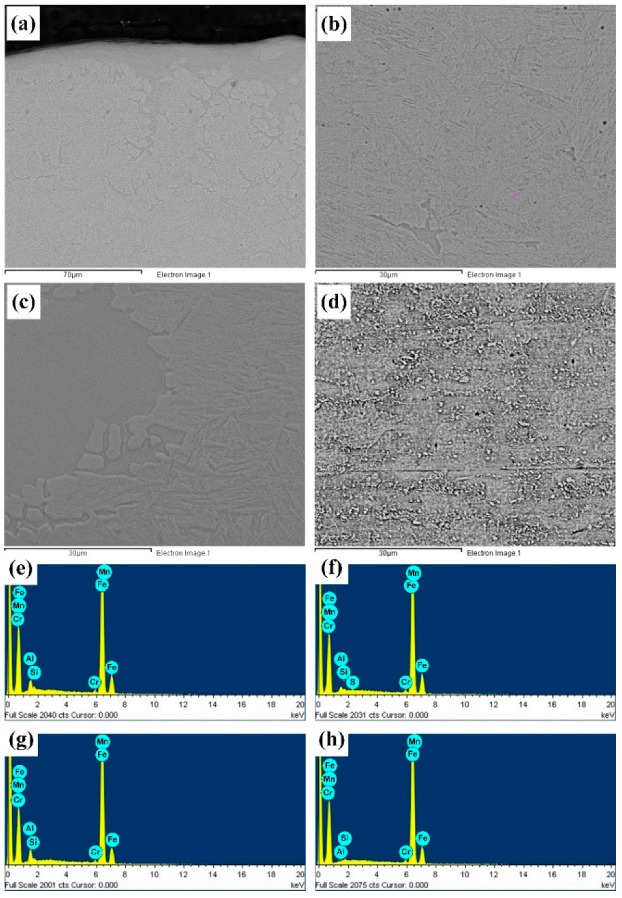
SEM cross-sectional morphologies and EDS (energy dispersive spectroscopy) spectrums for different areas of laser welding joint: (**a**) Top, (**b**) middle, (**c**) bottom, and (**d**) base material.

The Al-Si coating was fused into the weld after melted, and distributed throughout the whole weld. The distribution state of Al and Si elements in the entire weld and base metal were tested by an energy dispersive spectrum analyzer. The elemental compositions of the upper, middle, and lower parts of welds, and base material were tested, the results are shown in Figure 8 and Table 3.

It can be seen from Figure 8 and Table 3 that the base material itself did not contain the Al element, and the content of Si element was significantly lower than that in the welding zone. Before solidification, the weld pool was in a state of continuous flow. The Al-Si coating was fusing into the weld after melted. Al elements in the weld were flowing along with the weld pool, and drawn into each zone of weld. Therefore, Al elements were found in the upper, middle, and lower welds. Under the current technology, the complete penetration of Al-Si coated HPF steel was available, so the Al-Si coating on the lower surface of test specimen was also fused and carried into the weld along with the flow of the weld pool. Due to the rapid heating and cooling characteristics of laser welding, only a part of Al and Si elements were drawn into the middle of the weld, while the remaining most Al and Si elements were left at the upper and lower parts. Therefore, the upper and lower parts of weld became the enrichment zones of Al and Si elements, while those in the middle part were less.

In the metallographic and SEM images, the bright white corrugated phases were found in the middle of the weld. It was presumed that the Al-Si coating melted and flowed into the weld, contributing the formation of Fe-Al intermetallic compounds or solid solutions. Analysis was conducted to explore specific compositions.

**Figure 9:**
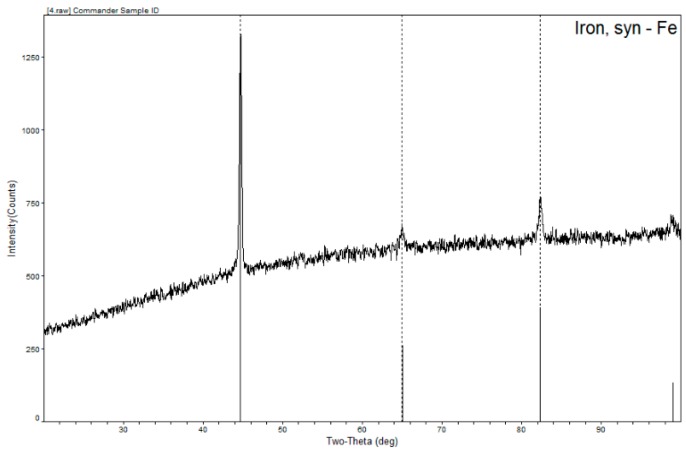
XRD patterns of the Al-Si coated HPF steel laser welded joint.

After the analysis of XRD pattern of Al-Si coated HPF steel laser welded joint, as shown in Figure 9, only the corresponding diffraction peak of Fe element was discovered, but no peak of the new phase could be found. This might be due to its detection accuracy. XRD had lower accuracy in identifying a small number of phases. Therefore, TEM was used for further identification in the following analysis.

**Figure 10:**
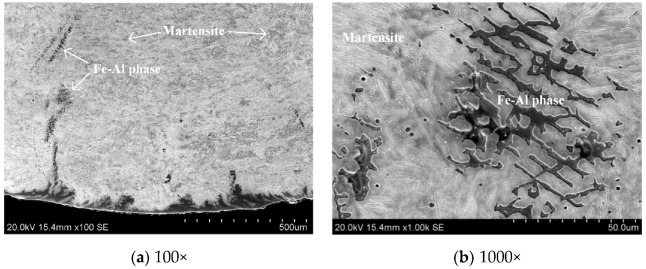
SEM morphologies of the Fe-Al phase distributed in the weld zone.

The Fe-Al phase generated was observed by 1000× SEM, as shown in Figure 10. It was found that the Fe-Al phase was distributed among reticulated epitaxial crystals. The microstructure of weld zone was originally uniform martensite. However, the mechanical properties of the weld was reduced due to the emergence of Fe-Al phase and uneven microstructure. 

Under the action of external loads, the dislocation in some grains started first and accumulated at the grain boundary. When the external loads continued, the adjacent grains received and reached the dislocation through the grain boundary, and then the dislocation of adjacent grains started, thus resulting in plastic deformation. The matrix phase was hard and brittle, and the grain boundary had a strong ability to hinder dislocation movement. While the Fe-Al phase was relatively soft to the matrix, it was prone to undergo plastic deformation. There was a dislocation ring around the Fe-Al phase particle. When the applied load is large to a certain extent, the dislocation ring will move towards the Fe-Al phase particle and forms a microvoid. The crack was easy to form a nucleus here, thus forming a microcrack, and then spreading and breaking.

**Figure 11:**
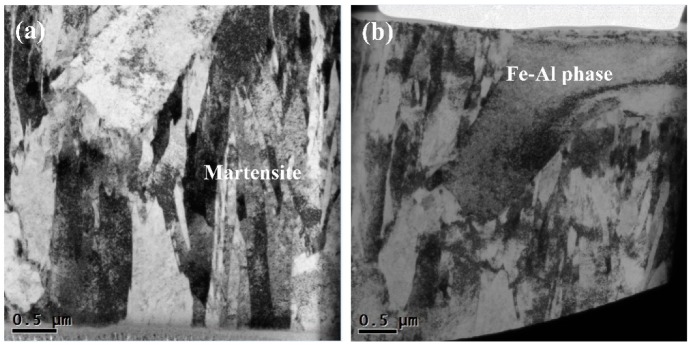
TEM bright field images of the weld.

In order to determine the specific image of this Fe-Al phase, TEM microscopic analysis was performed on the specimen. The obtained results are shown in the Figure 11. It can be seen that most of the microstructures in the figure were laminated low carbon martensite, roughly in the same size, and directional parallel arrangement, and the substructure was a high-density dislocation network. In the middle of the specimen, two grains completely different from martensite tissues were obviously lighter in color than martensite, and the grain size was larger, which was presumed to be the Fe-Al phase in the fusion weld during welding.

**Figure 12:**
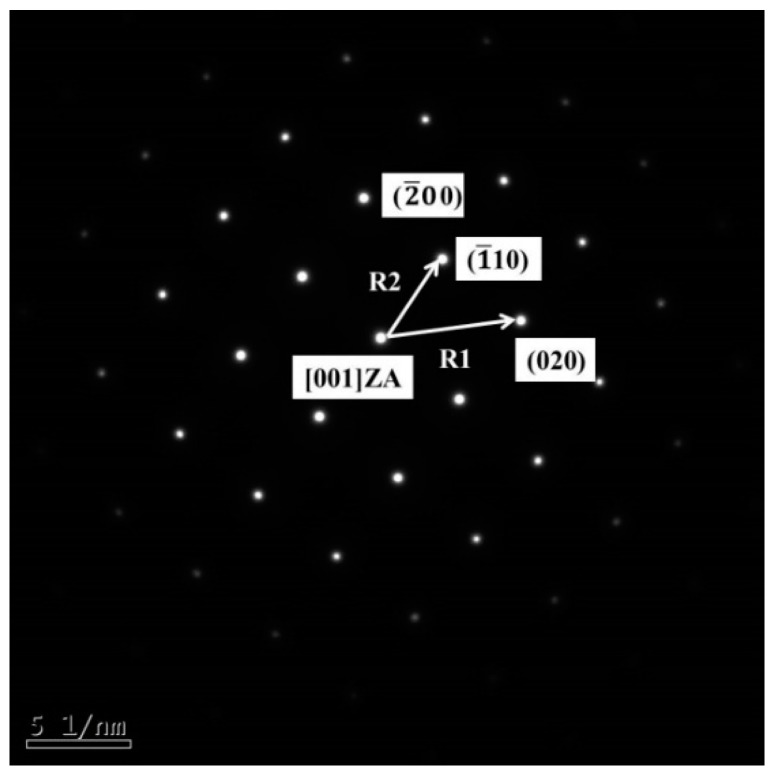
Selected electron diffraction pattern of the new phase.

The diffraction pattern of the new phase was observed, as shown in Figure 12. The four points, which were not collinear in the diffraction pattern, were calibrated, and the distance on the figure was measured. Among them, the crystal surface spacing of R1 was 0.1478 nm, and that of R2 was 0.2042 nm; after the comparison with diffraction pattern of α-Fe was made, R2/R1 = 1.4015, which was close to the calibration constant of 1.414, and then the four surrounding crystal planes were calibrated, as shown in Figure 12. The zone axis index was calculated to be [001].

Combined with the Fe-Al binary phase diagram, it could be obtained that when the atomic percentage of Al element was less than 20%, the corresponding phase was α-Fe, namely, the solid solution of Al in α-Fe. The solid solution is divided into interstitial solid solution and substitutional solid solution. For this kind of Fe-Al phase, since the atomic radius of Fe was 1.27 Å, the atomic radius of Al was 1.43 Å, and the atomic radius ratio of the both was 1.27/1.43 = 0.89. The radii of the two atoms were not much different, and the solid soluble Al atoms could not enter into the interstitial voids of α-Fe; therefore, only the substitution solid solution of Al could be formed in α-Fe at this time.

### 3.4. Hardness Values 

**Figure 13:**
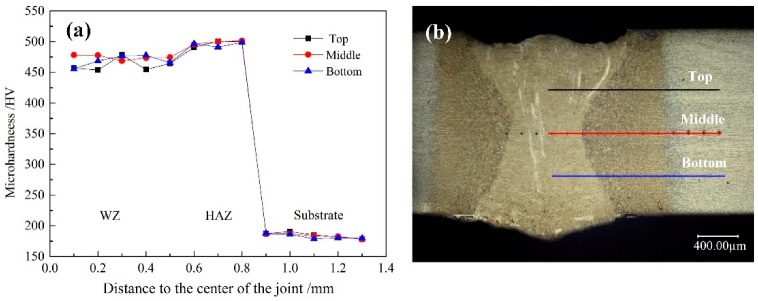
(**a**) Microhardness curve of welded joints, and (**b**) microhardness test positions.

Figure 13 shows the microhardness change curve of the base metal, HAZ, and weld zone of the laser weld of Al-Si coated HPF steel. It can be seen from the figure that the weld hardness was significantly different in each zone, in which the microhardness of weld zone and HAZ was significantly higher than that of the base metal. This was because of the rapid heating and cooling characteristics of laser welding, the cooling rate of metal in weld pool was faster when solidification occurred, and the base metal in the weld was completely changed into austenite, resulting in martensite. As the base metal did not experience thermal circulation, the main microstructure was still ferrite, while the martensite structure strength was significantly higher than that of ferrite, so the hardness of weld zone and HAZ was significantly higher than that of base metal.

Compared with the microhardness of the weld zone, the microhardness of HAZ could be improved slightly. This is because the welding edge was far away from the heat source, and the cooling rate was higher than the weld center, so the martensite microstructure generated was smaller and stronger. It can be seen from Table 4 that the maximum hardness in the middle of the weld was 484.4 HV, higher than that in the upper and lower parts of the weld. This was because the Fe-Al phase in the middle of the weld was less than the upper and lower parts of the weld, while the Fe-Al phase was a weakened phase. The hardness test of this phase shows that its hardness was 330.7 HV, which was significantly lower than other parts of the weld. Therefore, its existence could reduce the average strength of weld joint. The content of this phase in the middle of weld joint was the least, the joint was affected by the least softening, so the hardness was the highest in the middle of weld.

**Figure 14:**
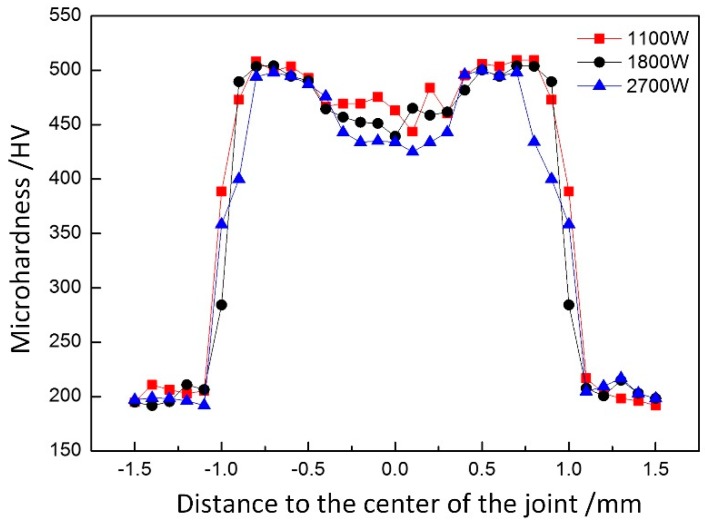
Microhardness distribution curve of welded joints at different laser powers.

The microhardness distribution curves of welding joints at different laser powers were compared, as shown in Figure 14. When the laser power was 1.1 kW, the microhardness of the weld zone was the highest, namely, 466.53 HV, and the minimum microhardness was 440.45 HV when the laser power was 2.7 kW. It could be found that the microhardness of weld zone and thermal zone decreased with the increase of laser power when other process parameters were maintained the same. This was because the increase in laser power slowed down the cooling rate of the weld joint, thus resulting in the larger martensite and lower weld hardness.

Through the analysis of hardness distributions of different weld joints, on the one hand, as the heat input increased, the grain size was increased, so the microhardness was reduced. On the other hand, with the formation of the Fe-Al phase, the hardness of the joint decreased as its hindering effect on dislocation was weaker than that of other microstructures in the weld joint. Moreover, due to its uneven distribution in the weld joint, the aggregation of upper and lower parts was obvious, but less in the middle, thereby resulting in the difference in hardness distribution.

### 3.5. Tensile Strength

**Figure 15:**
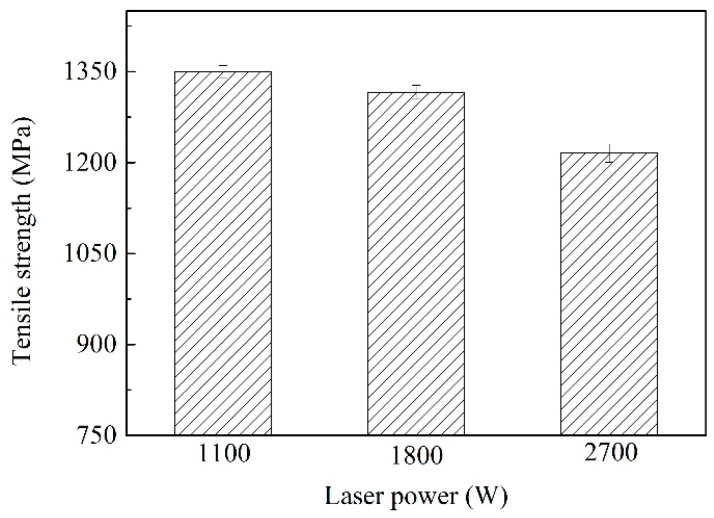
Tensile strength of the joint at different laser power.

As the laser power increased, the tensile properties of the welded joint were decreased. Figure 15 shows the tensile strength at different powers, when the laser power was 1.1 kW, the maximum tensile strength of the joint was 1349.97 MPa. The minimum tensile strength of weld was 1215.76 MPa when the laser power was 2.7 kW. This was because as the thermal input increased, the cooling rate of the entire joint decreased and the grain size at the joint position became larger. The larger the grain size, the less the total number of grains contained in the joint, which intensifies the segregation of impurities at the grain boundary. Under the action of external tensile load, the total number of grains that produces plastic deformation decreased, which aggravated the non-uniformity of the deformation of specimen, and the stress concentration was more easily generated in the entire joint. The inhibition effect of the grain boundary on the crack was obvious, when the grain size was large, the total area of grain boundary decreased relative to the grain size, and the degree of crack inhibition by the grain boundary also decreased. Therefore, the tensile strength of the joint decreased with the increase of thermal input.

**Figure 16:**
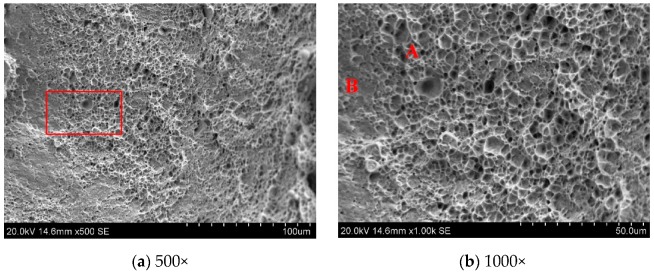
Typical tensile fracture morphologies of the joint laser welded at a laser power of 1.1 kW. (**a**) fracture surface of the laser welded joint; (**b**) magnified image of red box in (**a**).

SEM analysis of the tensile fractures shows that the fracture mechanism of the weld joint obtained by the three different laser powers was all subject to quasi-cleavage fracture. Under the action of external loads, the fiber area, radiation area, and shear lip area were usually found at the fracture site (Figure 16b). In this experiment, the shear lip area of fracture was not obvious. As shown in Figure 16b, there were many dimples in area A, and the cracks in this region formed nuclei and expanded the fiber area. The area B was smooth, and the dimples rarely showed the features of the transient fault area.

## 4. Conclusions

1. The stability of laser welding on the Al-Si coated HPF steel was poor, and metal steam generated due to the burning loss of Al-Si coating was observed. At a laser power of 1.1 kW and a welding speed of 1.5 m/min, the formation of weld was the best.
2. During the welding process, the Al-Si coating on the specimen surface was melted, and drawn into the weld. The generated Fe-Al phase was not uniformly distributed in the weld. According to selected area electron diffraction analysis, the Fe-Al phase was determined to be the solid solution of α-Fe and Al.
3. The hardness in the Fe-Al phase distribution zone was lower (330.7 HV). The hardness in the middle weld was 484.4 HV, higher than that in the upper weld (459.8 HV) and lower weld (461.4 HV). When the laser power was 1.1 kW, the highest average hardness in the weld zone was 466.53 HV.
4. As the laser power increased, the tensile property of weld showed a downward trend. When the laser power was 1.1 kW, the weld had the best tensile property, namely, 1349.9 MPa. Fracture pattern of welds was the quasi-cleavage fracture.

## Figures and Tables

**Table 1:** Laser Welding Process Parameters.

Parameter	Value
Laser power (kW)	0.9–2.7
Welding speed (m/min)	1.5
Focal length (mm)	250
Defocusing distance (mm)	−1
Type of shielding gas	Ar
Gas flow (L/min)	10–30

**Table 2:** Weld Sizes at Different Laser Powers.

Laser Power (kW)	Weld Penetration (mm)	Weld Width (mm)	Width of Heat Affect Zone (mm)
0.9	1.72	1.61	1.82
1.1	2	1.58	1.96
1.8	2	1.69	2.34
2.7	2	1.92	2.80

**Table 3:** Chemical Compositions of Different Areas of the Laser Welded Joint.

Locations	Al	Si	Mn	Fe
Top	4.35	1.35	1.46	92.70
Middle	1.93	0.74	1.69	92.83
Bottom	4.89	0.92	1.23	92.90
Base material	0.00	0.53	1.54	97.53

**Table 4:** Average Microhardness of Different Areas of the Joint.

Position	Top	Middle	Bottom	Base Material	Fe-Al Phase
Average value	459.8 HV	484.4 HV	461.4 HV	185.3 HV	330.7 HV
